# Highly specific Immunoproteasome inhibitor M3258 induces proteotoxic stress and apoptosis in KMT2A::AFF1 driven acute lymphoblastic leukemia

**DOI:** 10.1038/s41598-025-01657-0

**Published:** 2025-05-19

**Authors:** Tyler W. Jenkins, Jacquelyn Elise Fitzgerald, Jieun Park, Addison M. Wilson, Kristy L. Berry, Keith S. Wong, Walid A. Houry, Irene Lee, Andrey V. Maksimenko, Peter R. Panizzi, Yulia Y. Maxuitenko, Matthew Shane Loop, Amit K. Mitra, Alexei F. Kisselev

**Affiliations:** 1 Department of Drug Discovery and Development, Harrison College of Pharmacy, Auburn University, Auburn, AL 36849 USA; 2 Division of Research, Harrison College of Pharmacy, Auburn University, Auburn, AL 36849 USA; 3 Department of Health Outcomes and Research Policy, Harrison College of Pharmacy, Auburn University, Auburn, AL 36849 USA; 4 Department of Biochemistry, University of Toronto, 661 University Avenue, MaRS Centre, West Tower, Toronto, ON M5G 1M1 Canada; 5 Department of Chemistry, University of Toronto, Toronto, ON M5S 3H6 Canada; 6 Department of Chemistry, Case Western Reserve University, Cleveland, OH 44106-7078 USA; 7 Present Address: Vanderbilt University Medical Center, Nashville, TN USA; 8 Present Address: School of Medicine, University of Alabama at Birmingham, Birmingham, AL USA; 9 Auburn University, 720 S. Donahue Dr., Auburn, 36849-5503 AL USA

**Keywords:** Proteasome, Proteasome inhibitor, Ubiquitin, Proteostasis, Heat shock response, Unfolded protein response, Proteasome, Acute lymphocytic leukaemia, Target validation

## Abstract

Proteasome inhibitors (PIs) bortezomib, carfilzomib and ixazomib are approved for the treatment of multiple myeloma and mantle cell lymphoma and have clinical activity in acute lymphoblastic leukemia (ALL). The predominant form of proteasome in these hematologic malignancies is the lymphoid tissue-specific immunoproteasome. FDA-approved PIs inhibit immunoproteasomes and ubiquitously expressed constitutive proteasomes causing on-target toxicities in non-hematological tissues. Replacing PIs with selective immunoproteasome inhibitors (IPIs) should reduce these toxicities. We have previously shown that IPI ONX-0914 causes apoptosis of ALL cells expressing the KMT2A::AFF1 (MLL-AF4) fusion protein but did not elucidate the mechanism. Here we show that a novel, highly specific IPI M3258 induces rapid apoptosis in ALL cells in vitro and is comparable to bortezomib in its ability to reduce tumor growth and to cause tumor regression when combined with chemotherapy in vivo. Treatment of KMT2A::AFF1 ALL cells with M3258, ONX-0914, and bortezomib induced proteotoxic stress that was prevented by the protein synthesis inhibitor cycloheximide, which dramatically desensitized cells to PI-induced apoptosis. Thus, similar to multiple myeloma, ALL cells are sensitive to PIs and IPIs due to increased proteotoxic stress caused by elevated rates of protein synthesis.

**Supplementary Information:**

The online version contains supplementary material available at 10.1038/s41598-025-01657-0.

## Introduction

Proteasome inhibitors (PIs) bortezomib (Btz, Velcade), carfilzomib (Cfz, Kyprolis) and ixazomib (Ixz, Ninlaro) are approved for the treatment of multiple myeloma (MM) and mantle cell lymphoma. Btz and Cfz have also shown clinical efficacy in acute lymphoblastic leukemia (ALL) in combinations with chemotherapy^1–5^. Clinical activity of PIs is limited by their toxicities, some of which, e.g. gastrointestinal and cardiac toxicities^6,7^, are caused by inhibition of proteasomes in non-malignant tissues.

There are two forms of proteasomes. The constitutive proteasome is expressed ubiquitously, while the interferon-γ inducible immunoproteasome^8^ is the predominant form of proteasome in the lymphoid tissues and in hematologic malignancies^9–11^. Btz, Cfz, and Ixz are equipotent inhibitors of constitutive proteasomes and immunoproteasomes^12,13^. Gastrointestinal toxicities of PIs and cardiac toxicity of Cfz are believed to be caused by inhibition of constitutive proteasomes in the gut and in the heart. Expression of immunoproteasomes in non-lymphoid tissues is low, raising the possibility of reducing on-target non-hematologic toxicities by replacing Btz and Cfz with selective immunoproteasome inhibitors (IPIs). The expression ratio of immuno- to constitutive proteasomes in ALL exceeds 10:1 and is the highest among hematologic malignancies^9–11^. Most ALLs are diagnosed in children. Btz inhibits bone growth and causes hypogonadism in young mice^14,15^, raising a concern about similar side effects in children. Replacing Btz with IPIs should reduce the risk of these pediatric-specific toxicities. Thus, ALL is the best hematologic malignancy to explore replacement of Btz and Cfz with IPIs.

Numerous inhibitors of immunoproteasomes have been developed^16^, and one of them, KZR-616 (zetomipzomib)^17^, is undergoing clinical trials for the treatment of lupus, where Btz has demonstrated clinical activity but was not developed further because of toxicity^18,19^. ONX-0914, a close structural analog of KZR-616, has activity in multiple murine models of autoimmune disease^20,21^. We have previously shown that ONX-0914 causes apoptosis of MM and ALL cells that express the KMT2A::AFF1 (MLL-AF4) fusion protein as the result of t(4;11)q(21;23) chromosomal translocation^22,23^. ONX-0914 also inhibited in vivo growth of orthotopic xenografts of ALL cells^23^.

M3258 is a novel, highly specific IPI, which was developed for the treatment of MM^24,25^, and has not been tested in ALL models. It is an orally bioavailable dipeptide boronate. KZR-616 and ONX-0914 are tetrapeptide epoxyketones, which are not orally bioavailable^17,20^. KZR-616 is dosed once weekly in human clinical trials. ONX-0914 has been dosed up to three times a week in mice^20^. Btz and Cfz are also administered once weekly as a bolus infusion^26^. Their concentrations and proteasome inhibition in blood peak within an hour after short infusion but decline rapidly, leading to complete recovery of proteasome activity within 24 h^26^. M3258 can be dosed daily in mice resulting in continuous suppression of immunoproteasome activity^24,25,27^. These differences between M3258, ONX-0914, and KZR-616 warrant a separate study of M3258 in cell culture and animal models of ALL.

Constitutive proteasomes and immunoproteasomes have three distinct pairs of active sites, which are the β5c (PSMB5), β2c (PSMB7) and β1c (PSMB6) in the constitutive proteasomes, and the β5i (PSMB8, LMP7), β2i (PSMB10, MECL1), and β1i (PSMB9, LMP2) in the immunoproteasomes. β5c and β5i are the prime targets of FDA-approved PIs^12,13^. These sites are responsible for the chymotrypsin-like activity, which is the most important for protein breakdown^12,16^. β2i and β2c are responsible for the trypsin-like activity, and β1c and β1i are responsible for the caspase-like activity. M3258, ONX-0914, and KZR-616 are very potent inhibitors of the β5i sites^20,24^ but differ in their ability to co-inhibit other sites. KZR-616 inhibits the β1i site^17^. ONX-0914 co-inhibits β1i and β5c sites at cytotoxic concentrations in vitro and at therapeutically effective doses in mice^20^. M3258 does not inhibit β1i, β2i, β1c, and β2c sites and is a weaker inhibitor of β5c sites than ONX-0914^24^. We have shown that continuous treatment with specific inhibitors of β5 sites causes apoptosis of cells expressing the constitutive proteasomes^28^. When cells are pulse-treated with PIs to mimic clinical exposure^26^, specific inhibition of the chymotrypsin-like sites is not sufficient to induce apoptosis, and co-inhibition of the caspase-like and the trypsin-like activities is required^13,29–31^. M3258, a once-daily orally-dosed compound, is the only inhibitor capable of continuous suppression of β5i activity in vivo^25^ that can be used to address whether continuous suppression of β5i activity is sufficient to block the growth of leukemia cells in vivo.

MM cells are highly sensitive to PIs because they are under constant endoplasmic reticulum (ER) stress due to the production of large amounts of immunoglobulins (IGs)^32–34^. Although activation of the unfolded protein response (UPR) has been reported at a specific stage of B-cell development^35^ and sensitivity of primary ALL cells to PIs was correlated with high basal levels of ER stress^11^, alternative mechanisms have also been proposed for cells that express the KMT2A::AFF1 fusion protein and are highly sensitive to Btz^36,37^.

In this study, we demonstrate that KMT2A::AFF1 expressing ALL cells are sensitive to M3258 in vitro and in vivo, that treatment with Btz and IPIs activate proteotoxic stress pathways, and that blocking protein synthesis dramatically desensitizes KMT2A::AFF1 ALL cells to PIs- and IPIs- induced apoptosis.

## Results

### Specific Inhibition of β5i sites is sufficient to induce apoptosis in Btz-sensitive ALL cells

**Fig. 1:**
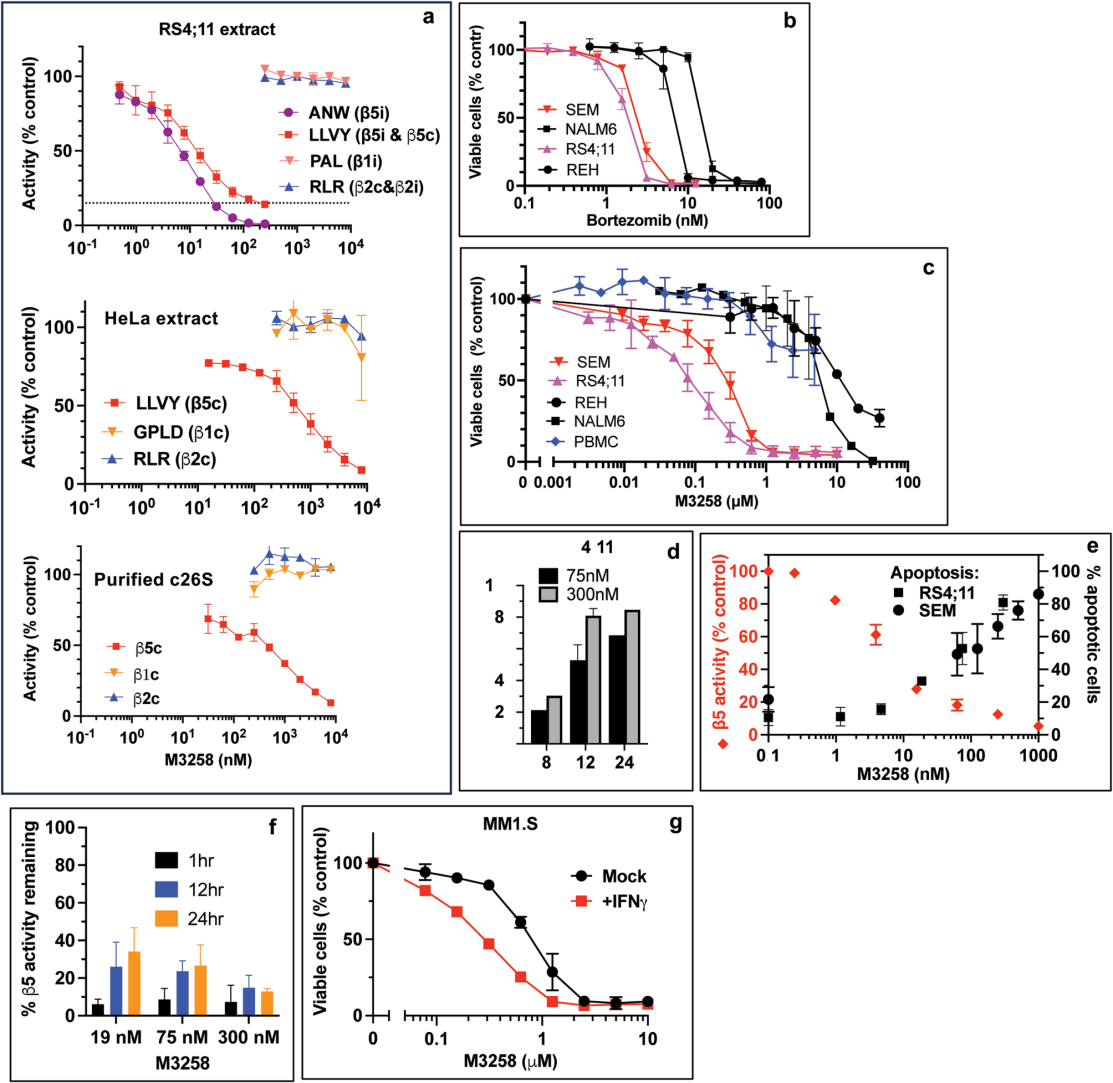
M3258, a highly specific inhibitor of β5i sites, induces apoptosis in ALL cells. (**a**) Inhibition of proteasome active sites was measured in extracts of RS4;11 and HeLa cells, and in the purified constitutive 26 S proteasomes; *n* = 2. (**b**, **c**) Cells were treated with Btz (b) or M3258 (c) for 48 h, and the viability was assessed by Alamar Blue; *n* = 2–4. (**d**) RS4;11 cells were treated with 75 nM and 300 nM M3258 for indicated times, and percentage of apoptotic cells was determined by flow cytometry; *n* = 2. (**e**) Cells were treated with M3258 for 12 h, and apoptosis was determined by flow cytometry. In a parallel experiment, RS4;11 cells were treated with M3258 for 4 h, and the chymotrypsin-like (β5) activity was measured by the Proteasome-Glo assay; *n* = 2. (**f**) The chymotrypsin-like (β5) activity of the proteasomes in cells treated with M3258 for times indicated was determined by the Proteasome-Glo assay; *n* = 2. (**g**) MM1.S cells were pre-treated with IFNγ for three days and then treated with M3258 for 48 h. Viability was measured with Alamar Blue; *n* = 2. Data on all panels are averages of n biological replicates, and the error bars indicate standard error of the mean.

To confirm M3258 specificity, we used fluorogenic substrates to measure inhibition of proteasomes in extracts of RS4;11 cells, which express ~ 7:1 ratio of immuno- to constitutive proteasomes^23^ and in the extracts of HeLa cells that express predominantly constitutive proteasomes^10^. We also measured inhibition of the purified constitutive 26 S proteasomes (Fig. 1a). M3258 inhibited cleavage of the β5i-specific substrate Ac-ANW-amc in extracts of RS4;11 cells with IC_50_ of ~ 10 nM and 150 nM M3258 completely blocked cleavage of this substrate. It also inhibited hydrolysis of Suc-LLVY-amc, which is cleaved by β5i and β5c, by ~ 85% confirming that immunoproteasomes are a predominant form of proteasomes in these cells. IC_50_ for Suc-LLVY-cleavage in extracts of HeLa cells, which express only β5c^10^, and by purified constitutive 26 S proteasomes was ~ 500 nM. In contrast to ONX-0914, Ac-PAL-amc cleavage by β1i sites was not inhibited by M3258 (Fig. 1a). β2i, β1c and β2c activities were also not inhibited by M3258. Thus, M3258 is a highly specific β5i inhibitor.

We conducted most of the experiments in RS4;11 and SEM cells that express the KMT2A::AFF1 fusion protein as the result of t(4;11)q(21;23) translocation, which were reported to be more sensitive to Btz than other ALL cells^36^. We confirmed that these lines are more sensitive to Btz than REH and NALM-6 ALL cells that do not express this protein (Fig. 1b). In contrast to our previous experiments where we pulse-treated cells with PIs and IPIs^22,23,38^, we treated cells with M3258 continuously because of its ability to cause a lasting suppression of immunoproteasome activity in vivo^25^. Btz-sensitive RS4;11 and SEM cells were also more sensitive to M3258 than REH and NALM-6 cells. Importantly, M3258 caused a minimal reduction of the peripheral blood mononuclear cells (PBMC) viability (Fig. 1c). Progressive reduction of viability of SEM and RS4;11 cells was observed at 0.1-1 µM M3258, which fell within the range of concentrations (0.1-6 µM) measured in the blood of mice that were treated daily with a safe 10 mg/kg dose of M3258^25^. Apoptosis was detectable 8 h after treatment and peaked at 12 h (Fig. 1d). About 50% of cells underwent apoptosis at the β5i-specific 100 nM concentration (Fig. 1e). Rates of apoptosis increased to 80% in cells treated with higher M3258 concentrations (Fig. 1e), which maintained inhibition for a longer time than lower concentrations (Fig. 1f). Thus, M3258 induces apoptosis in ALL cells at the β5i-specific and pharmacologically relevant concentration.

M3258 is a boronate. Boronates inhibit mitochondrial ATP-dependent serine proteases ClpP^39^ and LonP^40–43^, which are involved in the mitochondrial protein quality control. ClpP has been implicated in cancer^44–46^. Inhibition of ClpP could potentially contribute to cytotoxicity of M3258 but we found that M3258 does not inhibit human ClpP (Supplementary Fig. S1a). 2 µM M3258 inhibited LonP by only ~ 10%, while 2 µM Btz caused > 90% inhibition (Supplementary Fig. S1b). To further confirm that immunoproteasome is a target of M3258, we treated MM1.S cells, which express equal ratios of immuno- to constitutive proteasomes^22^, with interferon γ to increase expression of immunoproteasomes^8^. This treatment increased M3258 sensitivity (Fig. 1g). Thus, inhibition of β5i activity is the main cause of M3258 cytotoxicity.

Inhibition of another mitochondrial protease HtrA2/Omi is considered the cause of peripheral neuropathy in Btz-treated patients^47^. M3258 did not inhibit degradation of FITC-casein by HtrA2 (Supplementary Fig. S1c), further supporting the idea that M3258 may be a safer alternative to Btz.

### M3258 is active in animal models of ALL

**Fig. 2:**
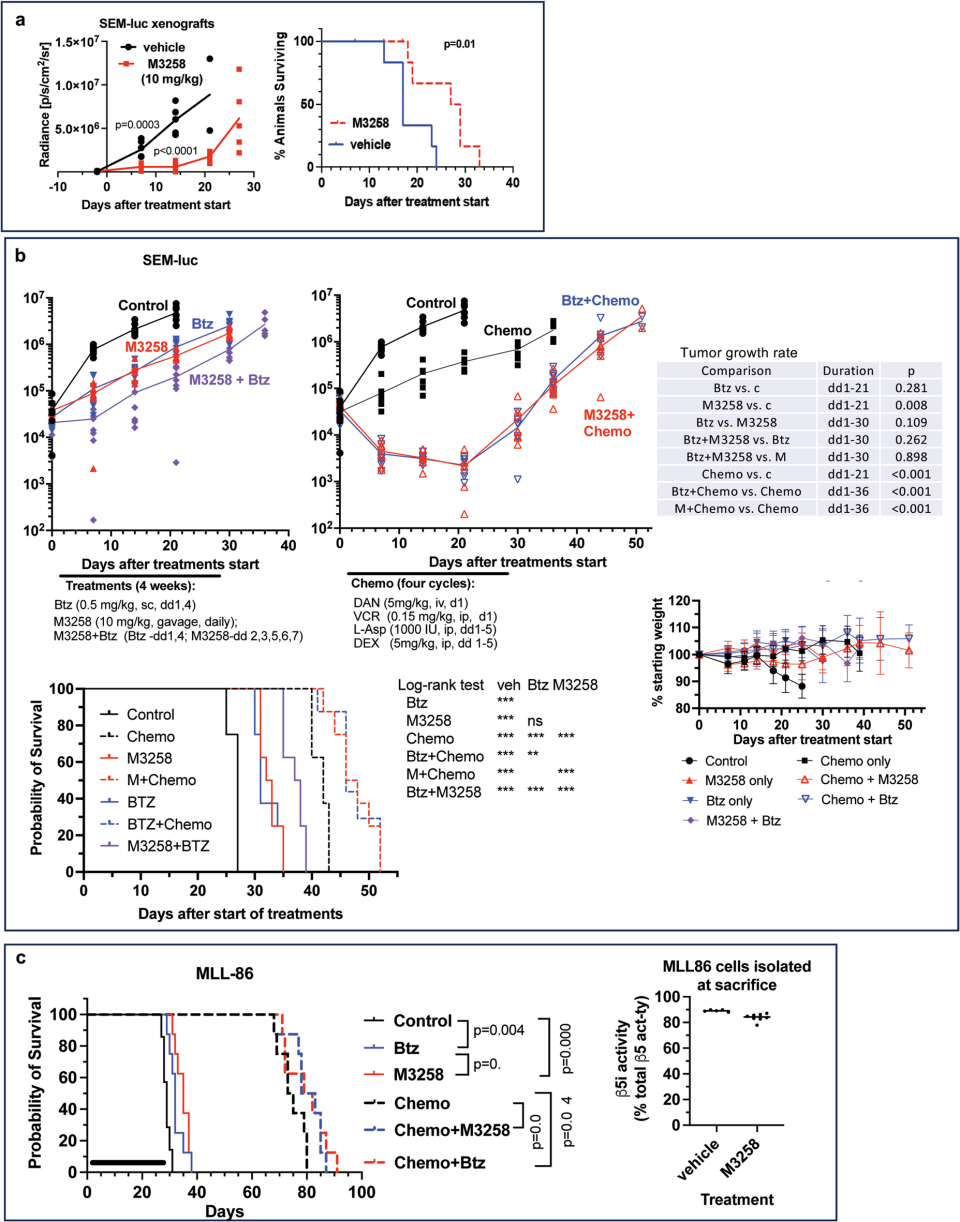
In vivo activity of M3258 in ALL models. (**a**) NSG mice bearing orthotopic tumors of luciferase expressing SEM cells were dosed daily by oral gavage for four weeks with either M3258 (10 mg/kg) or vehicle control, and tumor growth was assessed by bioluminescent imaging. Statistical analysis of growth data was conducted by a t-test; *n* = 6 (control); *n* = 7 (M3258-treated). Survival data here and in all subsequent panels was analyzed by the log-rank test. (**b**) NRG mice bearing the same tumors were treated as indicated and tumor growth was assessed by bioluminescent imaging. Left and right graphs present results of the same experiment. Slopes of log_10_(tumor growth) curves were estimated using linear mixed model with a random intercept for each mouse (Supplementary Table S2), and growth rates were compared using Bonferoni correction; *n* = 8. (**c**) NRG mice engrafted with MLL-86 cells were treated with Btz, M3258, and the same combination chemotherapy at the same schedule and doses as in panel b; *n* = 8. Left panel, survival of animals. Right panel, contribution of β5i to the chymotrypsin-like activity of proteasomes in 85–95% pure MLL-86 cells isolated from animals’ spleens.

We tested whether M3258 reduces growth of orthotopic xenografts of luciferase expressing SEM cells where tumor growth was measured by bioluminescent imaging (Fig. 2a). Survival of the animals, defined as the day when they reach protocol-defined end points, was the secondary readout. We used a once-daily 10 mg/kg dose of M3258, which previously showed activity in MM xenografts^24,25^. This treatment significantly delayed tumor growth and increased survival (Fig. 2a).

Next, we compared M3258 with Btz, and tested whether Btz and M3258 synergize in this model because we found that Btz and M3258 synergize in vitro in ALL cell lines (Supplementary Fig. S2a) and that ONX-0914 and Btz synergize in xenograft models of MM^22^. We used a twice-weekly 0.5 mg/kg dose of Btz, which is a murine equivalent of the human 1.5 mg/m^2^ dose. M3258 and Btz showed similar ability to inhibit tumor growth and increase survival (Fig. 2b, top and bottom left panels). Combining them with each other did not significantly delay tumor growth but improved survival compared with the single-agent treatments. Thus, M3258 and Btz have similar in vivo activity.

Since Btz and Cfz enhance the effect of chemotherapy in ALL patients^1–5^, this experiment also included comparison of M3258 and Btz in combination with the standard chemotherapy, consisting of vincristine (VIN), daunorubicin (DAN), dexamethasone (DEX) and L-asparaginase (L-Asp). We used dosing and schedule of vincristine, daunorubicin, dexamethasone and L-asparaginase that were previously shown to match their clinical pharmacokinetics^48^. Combinations of Btz and M3258 with chemotherapy caused tumor regression that lasted for 21 days without additional weight loss (Fig. 2b, upper right panel). Although tumors grew faster than in vehicle-treated group in subsequent weeks, adding Btz and M3258 to chemotherapy prolonged survival (Fig. 2b, bottom left). Adding M3258 or Btz did not lead to additional weight loss (Fig. 2b, bottom right). Importantly, M3258 and Btz have similar ability to cause tumor regression when combined with the chemotherapy.

We then compared M3258 and Btz in a patient derived xenograft (PDX) model. From a panel of 8 PDX models that express KMT2A::AFF1 fusion proteins^49^, we have chosen MLL-86, which was the most Btz-sensitive ex vivo (Supplementary Fig. S2b) and had a high enough ratio of immunoproteasomes to constitutive proteasomes (~ 7:1) to warrant M3258 testing (Fig. 2c, right panel). M3258 and Btz caused only modest increase in survival and did not provide significant benefit when added to the chemotherapy (Fig. 2c, left panel).

It has been reported previously that PBMCs and normal T-cells respond to the treatment with IPIs by replacing immunoproteasome with constitutive proteasomes^50,51^. Such scenario could lead to the rapid development of resistance. Therefore, we compared β5i contribution to the total chymotrypsin-like activity in MLL-86 cells isolated from mouse spleens of mock and M3258-treated animals. Although β5i contribution decreased slightly from 89 to 84%, most proteasomes were still immunoproteasomes (Fig. 2c, right panel). Thus, leukemia cells do not respond to M3258 treatment by replacing β5i with β5c.

### Co-treatment with specific inhibitors of the trypsin-like site and caspase-like sites increases M3258 cytotoxicity

**Fig. 3:**
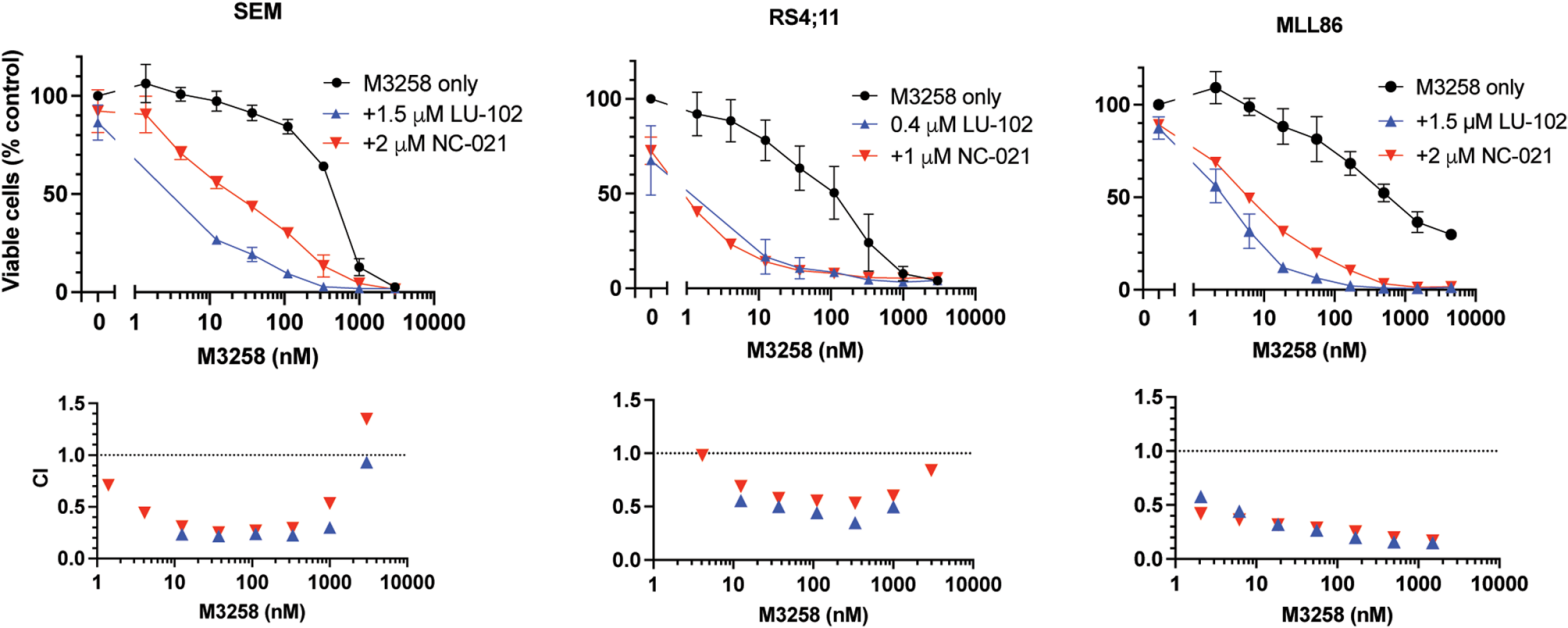
Specific inhibitors of the proteasome trypsin-like and the caspase-like sites enhance M3258 cytotoxicity. RS4;11 and SEM cells were treated with M3258 and indicated concentrations of the trypsin-like sites inhibitor LU-102 or the caspase-like sites inhibitor NC-021 for 48 h, and cell viability were measured with Alamar Blue. MLL-86 cells were treated ex vivo for 24 h, and cell viability was measured by the CellTiter Glo assay. All data are averages of two biological replicates. Error bars indicate the standard error. Single agent activity of NC-021 and LU-102 is presented on Supplementary Fig. S3 and in Fig. 3d in^23^. Bottom graph shows combination indexes determined by the CalcuSyn software.

Moderate activity of M3258 against KMT2A::AFF1 ALL in vivo has prompted us to ask whether it will be enhanced by inhibitors of the trypsin-like and the caspase-like sites, which were shown in our previous work to sensitize cells derived from various cancers to Btz, Cfz and ONX-0914^22,23,29–31,52^. Therefore, we tested whether LU-102, a specific inhibitor of the trypsin-like sites^52^, or NC-021, a specific inhibitor of the caspase-like sites^29^, increased cytotoxicity of M3258. Both inhibitors dramatically increased M3258 cytotoxicity in RS4;11 and SEM cells and in ex vivo cultured cells from MLL-86 model (Fig. 3). Thus, co-inhibition of either the trypsin-like or the caspase-like sites increases cytotoxicity of β5i-specific inhibitors.

### Mechanisms of ALL sensitivity to proteasome inhibitors

MM cells are highly sensitive to PIs because they are under constant proteotoxic stress due to production of large amounts of IGs^32–34^. Although activation of the UPR has been correlated with ALL response to PIs^11^, different mechanisms have been proposed for ALL cells that express KMT2A::AFF1 fusion protein and are highly sensitive to Btz^36^. It has been suggested that up-regulation of KMT2A::AFF1 upon treatment with PIs is sufficient to turn it into a pro-apoptotic protein^36^, and we confirmed that treatment of cells with M3258 causes MLL-AF4 upregulation (Supplementary Fig. S4). However, other studies have shown that overexpression of this protein causes growth arrest but not apoptosis^53,54^. PI-induced cell cycle arrest in the presence of apoptosis inhibitors has been attributed to the accumulation of a p27-cdk inhibitor, which is a transcriptional response shown to be dependent on KMT2A::AFF1 interactions with a B-cell transcription factor PAX5^36^. A recent study by Kamens et al. proposed a different explanation for the high Btz sensitivity of KMT2A::AFF1 expressing ALL cells. They found that Btz downregulated expression of many KMT2A::AFF1-dependent genes^37^, and the decrease was attributed to Btz-induced deubiquitylation of histone H2B, and subsequent decrease of histone H3 lysine-79 (H3K79) methylation. It is not known whether IPIs induce the same changes.

**Fig. 4:**
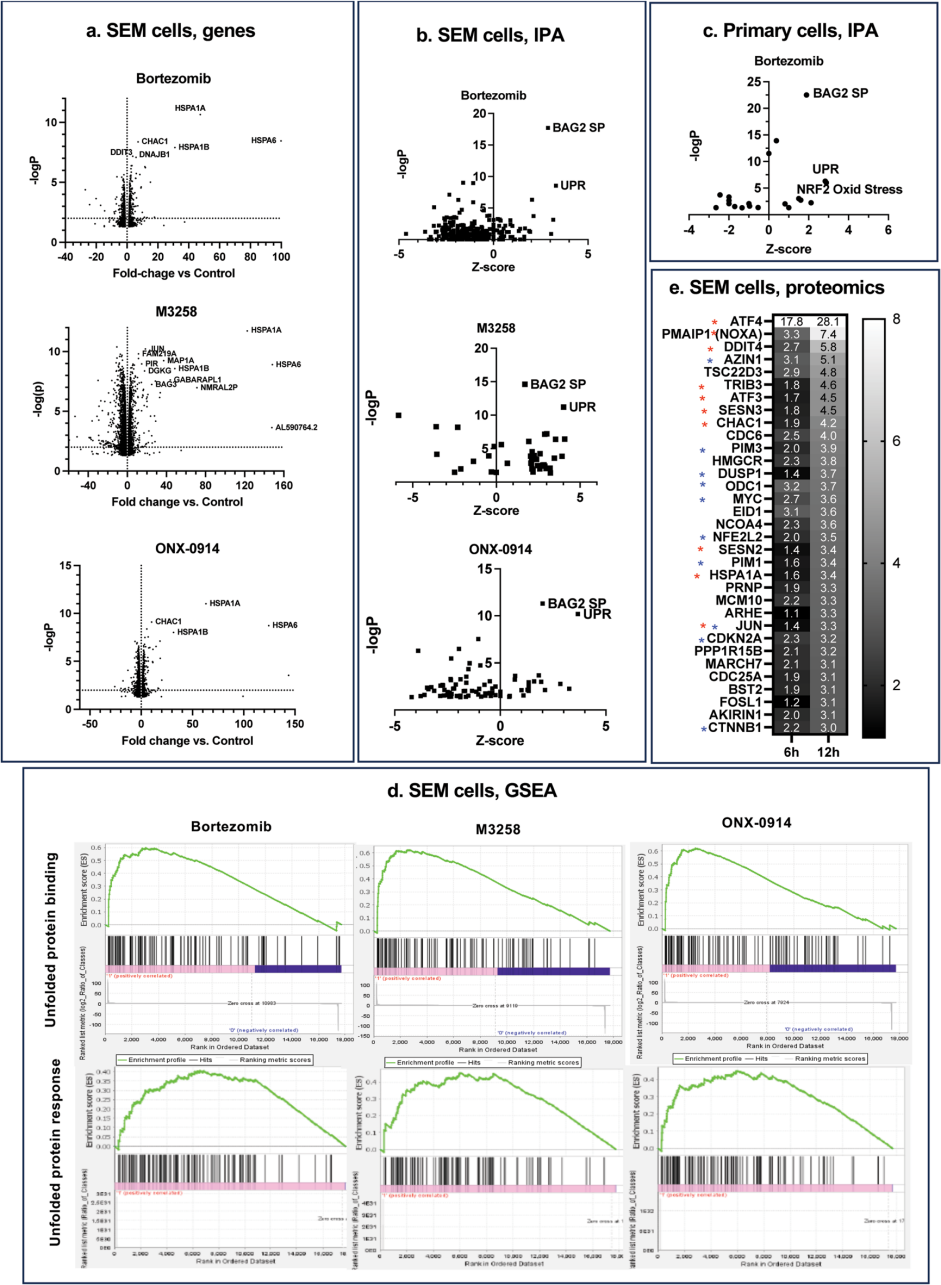
Treatment with PIs induces proteotoxic stress. (**a**) Effect of PIs on gene expression in SEM cells. Cells treated with 20 nM Btz, 1 µM M3258, and 0.8 µM ONX-0914 and harvested for RNA isolation 1 h before detectable increase in the caspase-like activity. (**b**) Analysis of RNA sequencing data from (a) by the Ingenuity Pathway Analysis (IPA). BAG2 SP, BAG2 signaling pathway. (**c**) IPA of RNA sequencing data of Btz-treated primary ALL cells from^37^. (**d**) Gene-set enrichment analysis (GSEA) of data from (a). (**e**) Analysis of published proteomic data from Btz-treated SEM cells^37^.

To gain insight into the mechanisms of PI and IPI-induced apoptosis in an unbiased fashion, we performed gene expression profiling of M3258, ONX-0914, and Btz-treated SEM cells. The mRNA encoding Hsp70 family of molecular chaperones, HSPA6, HSPA1A, and HSPA1B were the most upregulated by all three inhibitors (Fig. 4a). Ingenuity pathway analysis further revealed that UPR and BAG2 pathways were the most upregulated by all three compounds (Fig. 4b). BAG2 is a co-factor of the Hsp70 family of chaperons^55–57^, and BAG2 pathway contains HSPA6, HSPA1A, and HSPA1B and other cytosolic molecular chaperone genes. Analysis of the published data from Kamens et al.^37^ revealed that BAG2 is the most upregulated pathway in Btz-treated primary cells that carry the t(4;11) translocation (Fig. 4c). Gene set enrichment analysis (GSEA) revealed enrichment of UPR genes and genes that bind to unfolded proteins (e.g., molecular chaperons) in Btz-, M3258- and ONX-0914-treated SEM cells (Fig. 4d). Our analysis of proteomic data from Btz-treated SEM cells in the Kamens et al. study^37^ revealed that proteins upregulated by proteotoxic stress (ATF4, ATF3, DDIT4) are upregulated by Btz, alongside with several short-lived proteins (e.g., antizyme (AZN1), ornithine decarboxylase (ODC), myc, and HIF1α, Fig. 4e). Similar changes were observed in PI-treated MM cells^58,59^. Treatment with Btz also upregulated BH3-only protein NOXA, which is a key mediator of Btz-induced apoptosis^60–63^. Thus, PIs induce apoptosis in MM and ALL cells by similar mechanisms.

**Fig. 5:**
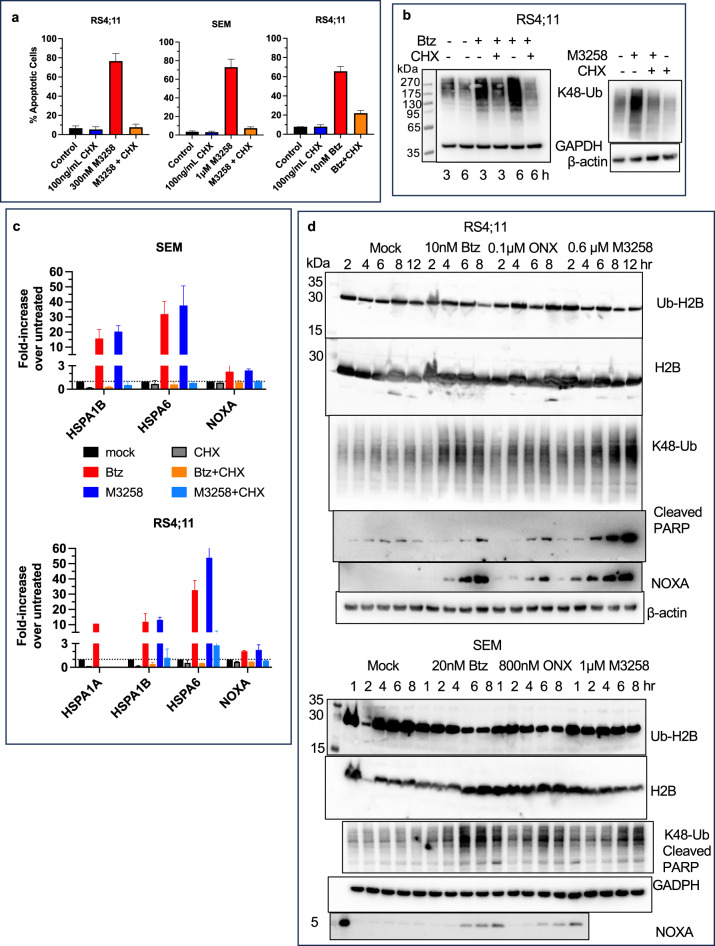
Blocking protein synthesis inhibits PI and IPI-induce apoptosis. (**a**) Cells were treated with M3258 for 12 h or Btz for 6 h in the presence or absence of cycloheximide (CHX), and apoptosis was measured by flow-cytometry with a caspase-3/7 probe; *n* = 2. Error bars indicate standard error. (**b**) RS4;11 cells were treated with 10 nM Btz for times indicated or with 1 µM M3258 for 6 h, in the presence or absence of 100 µg/ml CHX and analyzed by western blot. The left membrane was first incubated with K48-polyubiquitin antibody, and then with GAPDH antibody. The membrane on the right was cut horizontally and the top portion was incubated with K48-polyubiquitin antibody, and the bottom with β-actin antibody. (**c**) Cells were treated with either 10 nM Btz for 4 h or 1 µM M3258 for 6 h, in the presence or absence of 100 ng/ml CHX. RNA was isolated and analyzed by RT-Q-PCR; *n* = 2(RS4;11) or 3(SEM). Error bars indicate standard error. (**d**) Cells were treated with Btz, ONX-0914 and M3258 for times indicated, and isolated histones and cell extracts were analyzed by western blots. Histone blots (top two panels) were simultaneously probed with mouse H2B and rabbit H2B-Ub antibodies, then with fluorescently labeled secondary antibodies, and imaged on different channels. Other blots were cut horizontally, the top sections were first probed with cleaved PARP antibody, and then with K48-polyubiquitin antibody. The middle sections were probed with the loading control antibodies, The bottom section of the RS4;11 membrane was probed with NOXA antibodies. SEM samples probed with NOXA antibodies were run on a separate gel.

MM cells are highly sensitive to PIs because they are under constant proteotoxic stress caused by the synthesis of the large amounts of IGs^32–34^. Similar to myeloma^64^, treatments of KMT2A::AFF1 expressing cells with subtoxic concentration of CHX, which inhibit ~ 80% of protein synthesis (Supplementary Fig. S5), blocked M3258- and Btz-induced apoptosis (Fig. 5a). CHX also reduced Btz and M3258-induced accumulation of ubiquitylated proteins (Fig. 5b) and blocked transcriptional upregulation of HSP1A genes and NOXA (Fig. 5c), making it highly unlikely that effect of CHX was caused by the inhibition of synthesis of a specific pro-apoptotic protein(s). Thus, similar to MM, ALL cells are highly sensitive to PIs and IPIs because of proteotoxic stress created by the synthesis of nascent polypeptides.

If the inhibition of degradation of nascent polypeptides is the primary cause of sensitivity of ALL cells to M3258 and Btz, accumulation of proteasome substrates should precede induction of apoptosis. We found that Btz and M3258 induced accumulation of proteins, marked for proteasomal degradation by K48-linked polyubiquitin chains (Fig. 5d). Accumulation of ubiquitylated proteins preceded induction of NOXA and cleavage of PARP (Fig. 5d). On the other hand, histone H2B de-ubiquitylation, which was previously considered the major cause of Btz-induced apoptosis^37^, occurred after PARP cleavage and NOXA induction in Btz-treated cells. Furthermore, M3258 did not cause H2B de-ubiquitylation, and ONX-0914 reduced H2B ubiquitylation in SEM but not in RS4;11 cells (Fig. 5d). Thus, PI-induced apoptosis in KMT2A::AFF1 expressing cells is caused by the inhibition of overall protein degradation and not by the induction of histone de-ubiquitylation.

## Discussion

To the best of our knowledge, this study represents the first report of the activity of the novel highly specific immunoproteasome inhibitor M3258 in ALL. In contrast to ONX-0914, which was less active than Btz in SEM-luc model^22^, M3258 and Btz had comparable activity. It should be noted that Cfz was reported to not have activity in PDX models of ALL that express the KMT2A::AFF1 fusion protein^65^, although the 2 mg/kg dose used in that study was much lower than the 9 mg/kg murine equivalent of the commonly used human dose of 27 mg/m^2^. M3258 should be less toxic than Btz or Cfz because it does not inhibit constitutive proteasomes and does not inhibit HtrA2, inhibition of which is responsible for Btz-induced peripheral neuropathy^47^. We did not conduct comprehensive toxicology assessment of M3258 in our work because such study has already been published^27^. One of the surprising observations of that study was that M3258, a potent inhibitor of the most important site, β5i, of proteasomes in the immune cells, had low hematologic toxicity. It can be explained by the replacement of immunoproteasomes with the constitutive proteasomes in IPI-treated PBMC and normal T-lymphocytes^50,51^. Such replacement in leukemia cells would result in the rapid development of resistance, but we were surprised to discover that immunoproteasome was still predominant in MLL-86 PDX cells after a 4-week treatment with M3258 (Fig. 2c). These differences in response between normal and malignant lymphocytes can potentially widen therapeutic window of IPIs.

Induction of apoptosis upon continuous treatment with M3258, a highly specific inhibitor of the β5i sites, extends our earlier observations that continuous treatment with a highly specific inhibitor of β5 sites is sufficient to induce apoptosis in cells that express constitutive proteasomes^28^. Continuous treatment with M3258 used in this study recapitulates in vivo exposure to this drug better than the pulse treatment, which was appropriate in the studies of Btz, Cfz and ONX-0914. Although M3258 concentration in the blood of mice treated with a safe 10 mg/kg dose decreases to 100 nM in 24 h from the initial peak of 6 µM^25^, we found that 100 nM is still capable of inducing apoptosis. Importantly, it was reported that β5i activity in MM tumors remains completely inhibited in mice dosed daily with 10 mg/kg M3258^25^.

Even if specific inhibition of β5i activity was sufficient to induce apoptosis in vitro, co-inhibition of the caspase-like or the trypsin-like activities increased M3258 cytotoxicity to a similar degree, by which it decreased viability of cells pulse-treated with β5-specific inhibitors^22,23,29–31,52^. We did not try these combinations in the in vivo experiments because of sub-optimal pharmacokinetic properties of LU-102 and NC-021 and lack of inhibitors of the caspase-like and the trypsin-like sites with better pharmacological properties.

This study was focused on KMT2A::AFF1 expressing ALL cells because it was reported that expression of this fusion protein sensitizes cells to Btz^36^. To gain a deeper insight into the mechanisms we conducted gene expression profiling and analyzed available gene expression profiling and proteomic data^37^. Strong activation of proteotoxic stress pathways by Btz and IPIs, and complete inhibition of Btz and M3258-induced apoptosis by CHX has led us to conclude that Btz and IPI sensitivity is caused by high rates of protein synthesis. Future work should be focused on whether stabilization of KMT2A::AFF1 of PI increases protein synthesis further and whether it is compensated by the integrated stress response^66–69^. Thus, the molecular basis of ALL sensitivity to PIs is similar to MM, and solid tumors that express PTEN mutations, which lead to the activation of protein synthesis through the mTOR pathway^70–73^.

In the future, mechanisms of KMT2A::AFF1 upregulation should be studied. Although KMT2A::AFF1 is a short-lived protein, a dramatic upregulation seen on Fig. S4 and in previous studies^23,36^ may not be caused only by simple inhibition of its degradation. An upregulation of the transcription factor that drives KMT2A::AFF1 expression should also be considered. If KMT2A::AFF1 degradation is ubiquitin-dependent, the ubiquitin ligase should be identified. It has been reported that a reverse AFF1::KMT2A (AF4-MLL1) fusion protein is targeted for degradation by SIAH ubiquitin ligases^74^, but the binding site of these ligases is at the N-terminus of AFF1, which is absent in the AFF1::KMT2A fusion.

Our conclusion is different from those of Kamens et al. that KMT2A::AFF1-expressing ALL cells are highly sensitive to Btz because of Btz-induced histone H2B de-ubiquitylation, which leads to H3K79 demethylation and subsequent downregulation of KMT2A::AFF1 expressing genes^37^. In addition to our own gene expression profiling in SEM cell line, our analysis of their RNA sequencing data revealed that induction of proteotoxic stress was the most significant effect of Btz treatment of primary cells. They discovered a much more rapid and robust histone H2B de-ubiquitylation in cells treated with 50 nM Btz^37^ than what we observed in cells treated with 10–20 nM Btz, which is sufficient to kill most cells. We did not detect histone de-ubiquitylation until after the accumulation of ubiquitin conjugates, cleavage of PARP and induction of NOXA. Kamens et al. detected decreased expression of KMT2A::AFF1-expressing genes 20 h after treatment started, when, according to our data, most cells should have already undergone apoptosis. It is possible that extensive histone de-ubiquitylation observed by Kamens et al. was caused by overtreatment of cells with Btz. It is also possible that a small fraction of KMT2A::AFF1-expressing cells that survive apoptosis as the consequence of Btz-induced proteotoxic stress stop proliferating because of decreased expression of KMT2A::AFF1-dependent genes as the consequence of H2B de-ubiquitylation.

The biggest limitation of our study is that we conducted in vivo evaluation of activity in a PDX model, which turned out to not be very Btz sensitive, even though it was the most sensitive in the in vitro studies. This finding disagrees with the previous conclusion that expression of KMT2A::AFF1-protein can serve as a marker of response to Btz. It should be noted that although most ALL cells are highly sensitive to Btz in vitro^75,76^, in vivo response of the PDX models varied from a complete to a no response^77^ necessitating in vivo validation of all of the biomarker candidates, such as high basal levels of UPR activation and high ratio of immuno- to constitutive proteasomes^11^. Sensitivity of MM cells to PI depends on the proteasome load-to-capacity ratios^33,38^, and it should be explored in the future studies whether the same is true for ALL. In the absence of such markers, IPIs should be tested in PDX models of ALL that are known Btz responders^77^. Mechanism of acquired resistance to the combinations of chemotherapy with PIs and IPIs as seen in the SEM model would also be very interesting to address but limited funding has prevented us from asking all these interesting questions. ,

In summary, we have demonstrated that the novel IPI inhibitor M3258 is comparable to Btz in its anti-leukemia activity and that IPIs and Btz induce death of KMT2A::AFF1 expressing ALL cells through proteotoxic stress pathways.

## Materials and methods

### Inhibitors and substrates

Carfilzomib (Cat# B-3022) and bortezomib (Cat# B-1408) were obtained from LC laboratories. ONX-0914 (Cat# HY-13207), M3258 (Cat# HY-111790), and were obtained from MedChemExpress. Dexamethasone (NDC 13985-533-03, VetONE Cat# 501012), daunorubicin (NDC 42658-021021-02, HISUNUSA), vincristine sulfate (NDC 61703-309-16, HOSPIRA) and L-asparaginase (Merck, 7407114) were obtained from the Auburn University Veterinary Pharmacy. LU-102 (CAS#1421639-62-4) was kindly provided by Drs. Bogdan Florea and Herman Overkleeft (Univ. of Leiden, the Netherlands). NC-021 synthesis was described in^27^. Suc-LLVY-amc (7-amino-4-methylcoumarin) was obtained from Bachem (Cat #4011369). Ac-GPLD-amc was custom synthesized by Genscript; all other substrates were custom synthesized by China Peptide.

### Cell lines and cell culture experiments

HeLa S3 (RRID: CVCL_0058), RS4;11 (RRID: CVCL_0093), REH (RRID: CVCL_1650), and NALM-6 (RRID: CVCL_0092) cells were obtained from the American Tissue Culture collection; SEM (RRID: CVCL_0095) cells were obtained from DSMZ (Braunschweig, Germany). MM1.S (RRID: CVCL_8792) and GFP and luciferase (luc) expressing SEM cells were described in our previous study^23^. All cell lines were reauthenticated by short tandem repeat profiling (STR), conducted by BioSynthesis (Lewisville, TX) or LabCorp (Burlington, NC), at the end of the study. Peripheral blood mononuclear cells (PBMCs) were purchased from the Immune Monitoring Shared Resource of the Dartmouth Cancer Center. PDX cells were kindly provided by Dr. Richard Lock from Australian Children’s Cancer Institute and the Pediatric Pre-clinical Testing Program (PPTP)^49^. All lines were cultured in RPMI-1640 media supplemented with 10% FBS, penicillin, streptomycin, anti-mycoplasma antibiotic ciprofloxacin (0.2 µg/ml), and anti-mycotic agent amphotericin B (0.25 µg/ml). Cell viability was assayed with resazurin (Alamar Blue, Sigma). Combination indexes (CIs) were determined using CalcuSyn. PDX models were short-term cultured (24 h) in the same media, but only for the purpose of determining inhibitor sensitivity, and their viability after treatments was assessed by the CellTiter-Glo assay (Promega). Apoptosis was measured by flow-cytometry on BD Accuri C6 Plus flow-cytometer using CellEvent™ Caspase-3/7 Green Detection Reagent and SYTOX cell viability dye (ThermoFisher). Data were analyzed using BD CSampler Plus software.

### Measurement of proteasome Inhibition in extracts

Proteasome activity was measured in extracts of RS4;11 and HeLa S3 cells that were prepared by lysing frozen cell pellets in 50 mM Tris–HCl, pH 7.5, 25% sucrose, 2 mM EDTA, 1 mM DTT, 1 mM ATP, 0.05% digitonin, and subsequent centrifugation at 16,000 g for 15 min at + 4^o^C. Extracts (1 µg total protein/well) were added to a solution of a substrate and M3258 in 50 mM Tris-HCl, pH 7.5, 40 mM KCl, 1 mM EDTA, 1mM DTT, and 100 µM ATP. Cleavage of substrates was followed continuously on a fluorescent plate reader, and the rates were determined from the slope of the reaction progress curves^78^. We used the following fluorogenic substrates: Suc-LLVY-amc (chymotrypsin-like activity, β5c and β5i), Ac-ANW-amc (β5i), Ac-nLPnLD-amc (caspase-like activity, β1c and β1i), Ac-APL-amc (β1i) and Ac-RLR-amc (trypsin-like activity, β2c and β2i). All substrates were at 100 µM except for Ac-ANW-amc, which was at 200 µM. The same protocol was used to measure inhibition of constitutive 26 S proteasomes that were purified from human MDA-MB-231 cells as described^79^, which do not express immunoproteasomes. We did not preincubate extracts or proteasomes with M3258. To determine relative contribution of β5c and β5i to the cleavage of Suc-LLVY-amc assays were conducted in the presence of 100 nM M3258 because this concentration caused > 95% inhibition of β5i sites but did not inhibit the β5c sites (Fig. 1a).

### Measurement of proteasome inhibition in cells

The chymotrypsin-like (combined β5i and β5c) activity of proteasomes in intact inhibitor-treated cells was measured with the Proteasome-Glo assay (Promega) as described^31^.

### Histone isolation and western blot analysis

Frozen cell pellets were first lysed in a buffer of 50 mM Tris-HCl, 10% glycerol, 5 mM MgCl_2_, 1 mM EDTA, 1 mM ATP, and 10% CHAPS and centrifuged for 15 min at 16,000 g at + 4^o^C. The lysates were then used for western blot analysis. For histone isolation, the pelleted debris were resuspended in 400 µL of 0.4 N H_2_SO_4_ and incubated on a rotator for at least 30 min. Nuclear debris were removed by centrifugation for 10 min at 16,000 g at + 4^0^C. The supernatant was moved to a fresh tube, and 100% trichloroacetic acid (TCA) was added dropwise to a 33% final concentration. After 30 min on ice, histones were pelleted by centrifugation at 16,000 g at + 4^o^C for 10 min, washed twice with acetone, air dried for 20 min at room temperature, and dissolved in water. Histone concentrations and protein concentrations of the lysates were determined by Bradford (Coomassie Pus, Thermo Fisher), and used to normalize gel loading. Nuclear extracts for the analysis of KMT2A::AFF1 expression were prepared as described in our previous study^23^.

Cell extracts or purified histones were fractionated on a 10% Bis–Tris SurePAGE (Genscript) or NuPAGE (Thermo Fisher) using MOPS or MES running buffer and transferred to either an Immobilon-FL (if fluorescent secondary were used), or an Immobilon-PSQ (if enhanced chemiluminescence was used), or nitrocellulose (for KMT2A::AFF1 detection) membrane. Membranes were also probed with the following antibodies: mouse Histone H2B antibody (clone 53H3, Cell Signaling Cat#2934; RRID: AB_2295301), rabbit monoclonal Ubiquityl-Histone H2B(K120) antibody (clone D11, Cell Signaling Cat#5546; RRID: AB_10693452), K48-linkage specific polyubiquitin rabbit monoclonal antibody (clone D9D5, Cell Signaling Cat#8081; RRID: AB_10859893), NOXA rabbit monoclonal antibody (clone D8L7U, Cell Signaling Cat#14766; RRID: AB_2798602), cleaved PARP(D214) rabbit monoclonal antibody (clone D64E10, Cell Signaling Cat#5625; RRID: AB_10699459), β-actin mouse monoclonal antibody (clone 8H10D10, Cell Signaling Cat#3700; RRID: AB_2242334), GAPDH rabbit monoclonal antibody (clone 14C10, Cell Signaling Cat#2118; RRID: AB561053), GAPDH mouse monoclonal antibody (clone D4C6R, Cell Signaling Cat#97166; RRID: AB_2756824), puromycin mouse antibody (clone 12D10, Millipore-Sigma, Cat# MABE343; RRID: AB_2566826). The secondary antibodies were AlexaFluor647-conjugated goat anti-rabbit IgG antibody (Thermo Fisher Cat#A21245; RRID: AB_2535813), IRDye800 CW-conjugated goat anti-mouse antibodies (Li-COR Biosciences Cat#926-32210; RRID: AB_621842), HRP (horseradish peroxidase)-conjugated anti-rabbit (Cell Signaling Cat#7074; RRID: AB_2099233) and anti-mouse (Cell Signaling, Cat#7076; RRID: AB_330924) IgG. Super Signal West Femto Maximum Sensitivity substrate (Thermo Fisher) was used to detect HRP activity. All membranes were imaged on Azure c600 Imager. We used Broad Multicolor Pre-Stained Protein Standards (GenScript, Cat #M006240).

### RNA sequencing and pathway analysis

Untreated control and treated cells were harvested in RNAlater, high-quality RNA was isolated using QIAshredder columns and RNAeasy Mini kit (Qiagen) and stored at -80 °C. RNA quality assurance and control were performed using Nanodrop-8000 and Agilent 2100 Bioanalyzer. RNAseq libraries were constructed using Illumina TruSeq RNA Library Prep Kit v2 (poly A enrichment). NGS Libraries were size-selected, and RNA sequencing was performed on Illumina’s NovaSeq 6000 platform (150 bp paired-end) at > 20 million reads per sample. Sample preparation was conducted at the Auburn University Center for Pharmacogenomics and Single-Cell Omics (AUPharmGx), and high throughput sequencing was conducted by Novagene. RNAseq data analysis was performed using a combination of a command-line-based data analysis pipeline and Partek Flow Genomic Analysis Software. Briefly, raw fastq reads were pre-processed, mapped to the hg38 human genome, normalized to counts per million (CPM) values, and log2-transformed. Differential gene expression analysis was then performed using modified limma, an empirical Bayesian method, and genes with mean fold-change > |1| and *p* < 0.05 were considered significantly differentially expressed. These differentially expressed genes were used to identify top pathways by Ingenuity Pathway Analysis (IPA) and Gene Set Enrichment Analysis (GSEA) based on the MSigDB database enrichment background. The gene expression data has been archived in the NCBI Gene Expression Omnibus (GEO: GSE282470) repository.

### Q-PCR analysis

RNA was isolated using Trizol, and reverse transcribed using the SuperScriptTM III First-Strand Synthesis kit (ThermoFisher). Q-PCR was conducted using Bimake qPCR SYBR-Green Master Mix. Primers (obtained from IDT) are listed in the Supplementary Table S1. Gene expression was calculated relative to the β-actin reference gene by the Bio-Rad CFX Manager 3.1 software using ΔΔq analysis.

### Animal studies

6–8-week-old female NSG (NOD.Cg-*Prkdcscid Il2rgtm1Wjl*/SzJ, e.g. Nod/Scid/IL-2 receptor

γ-chain (RG) knockout, Jackson Lab stock number 005557) or NRG (NOD.*Rag1*^*null*^ *Il2rg*^*null*^, NOD/ RAGgamma/NOD/RG knockout, Jackson Lab stock number 007799) mice were injected intravenously with 1 million of luciferase and GFP expressing SEM cells (SEM-luc)^23^ or 2 million cells from MLL-86 PDX model^49^, and randomly assigned to treatment groups. Growth of SEM-GFP xenografts was monitored by weekly bioluminescent imaging. Mice were injected intraperitoneally with D-Luciferin (150 mg/kg in PBS) and imaged on Xenogen Lumina XRMS imaging system (PerkinElmer) under isoflurane anesthesia. Photon counts of the whole animal were determined using Living Image 4.7.2 software (Perkin Elmer). Treatment started approximately 10 days after engraftment when SEM-luc tumors were first detected by imaging. Engraftment of MLL-86 cells was followed by measuring human CD45-positive cells in the peripheral blood by flow cytometry using FITC-labeled human CD45 antibodies (clone 2D1, BD Biosciences Cat #345808; RRID: AB_400306) and FITC-labeled Mouse IgG1κ (clone MOPC-21, BD Biosciences Cat #555748; RRID: AB_396090) as an isotype control^80^. Treatment started approximately 10 days after engraftment when mean hCD45 + exceeded 1%. M3258 was formulated in 30% 2-hydroxypropyl-β-cyclodextrin in PBS and dosed orally through a gavage. Btz was formulated in PBS and dosed subcutaneously. Vincristine sulfate, dexamethasone and L-asparaginase were administered intraperitoneally, and daunorubicin was injected via the tail vein. All four chemotherapeutic agents were obtained from the pharmacy as injection-ready formulations. Doses and frequency are indicated on figures and/or in captions. Treatments continued for 4 weeks, and animals were then monitored until they reached protocol defined endpoints, at which point they were euthanized. Spleens were harvested from animals bearing MLL-86 xenografts, and human leukemia cells were isolated using Lymphoprep as described^80^. The purity of the preparation was assessed by flow cytometry using FITC-conjugated human CD45 antibodies as described above. All animal procedures were carried out according to the US Public Health Service Policy on Care and Use of Laboratory Animals. Experiments were approved by Auburn University IACUC (protocols 2020–3750 and 2023–5279). The results of the studies are reported according to ARRIVE guidelines.

### Statistical analysis

Except for the tumor growth data on Fig. 2b, which was analyzed using R version 4.3.0, statistical analysis was conducted using GraphPad Prism. Statistical test and sample size are described in figure legends.

## Electronic supplementary material

Below is the link to the electronic supplementary material.

Supplementary Material 1

## Data Availability

The RNA sequencing data are publicly available in the NCBI Gene Expression Omnibus (GEO: GSE282470) repository. Raw data from other experiments is available from the corresponding author upon request.
