# Supplementary material for: Highly specific Immunoproteasome inhibitor M3258 induces proteotoxic stress and apoptosis in KMT2A::AFF1 driven acute lymphoblastic leukemia
Source: Sci Rep. 2025 May 19;15:17284. doi: 10.1038/s41598-025-01657-0 (PMC12089620; doi:10.1038/s41598-025-01657-0)
Supplement: Supplementary file 1 — Supplementary Material 1 [file 41598_2025_1657_MOESM1_ESM.pdf]

Supplementary Data

Highly specific immunoproteasome inhibitor M3258 induces proteotoxic stress and apoptosis in MLL-AF4-driven acute lymphoblastic leukemia.

Tyler W. Jenkins[1,7], Jacquelyn Elise Fitzgerald[1,8], Jieun Park[2], Addison M. Wilson[1], Kristy L. Berry[1], Keith S. Wong[4], Walid A. Houry[4,5], Irene Lee[6], Andrey V. Maksimenko[1,9], Peter R. Panizzi[1], Yulia Y. Maxuitenko[1], Matthew Shane Loop[3], Amit K. Mitra[1], Alexei F. Kisselev[1]

[1]Departments of Drug Discovery and Development, [2]Division of Research, and [3]Department of Health Outcomes and Research Policy, Harrison College of Pharmacy, Auburn University, AL 36849, USA; [4]Department of Biochemistry, University of Toronto, 661 University Avenue, MaRS Centre, West Tower, Toronto, ON M5G 1M1, Canada; [5]Department of Chemistry, University of Toronto, Toronto, ON M5S 3H6, Canada; [6]Case Western Reserve University

Table S1. RT-Q-PCR primers.

Gene		Sequence
β-actin	FW	CCAACCGCGAGAAGATGA
	REV	CCAGAGGCGTACAGGGATAG
NOXA	FW	GAGATGCCTGGGAAGAAGG
	REV	TTCTGCCGGAAGTTCAGTTT
HSPA1A/B	FW	CTACCATTGAGGAGGTGGATTAG
	REV	CAAAGAAGTGAAGCAGCAAAGA
HSPA6	FW	CCCATCATTGAGGAGGTTGAT
	REV	CTGAAGGGCAGGATCATAGAAG

The annealing temperature for all primers was 60°C.

Table S2. Estimated slopes of $\log_{10}$(tumor growth) curves through the first 21 days.

Treatment	Slope	p-value[1]
Control	0.100	<0.001
BTZ Only	0.072	<0.001
Chemo + BTZ	-0.051	<0.001
Chemo + M3258	-0.062	<0.001
Chemo Only	0.052	<0.001
M3258 + BTZ	0.046	<0.001
M3258 Only	0.060	<0.001

[1]Adjusted p-value using Bonferroni correction.

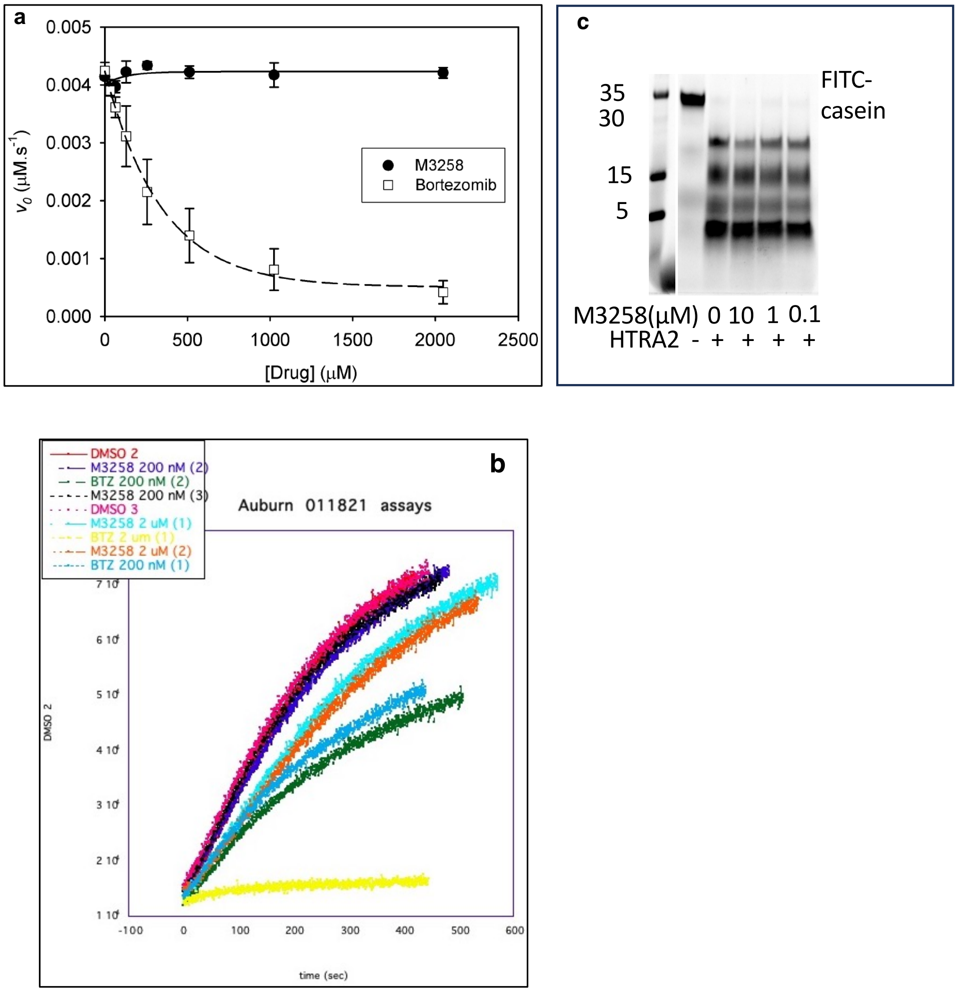

Figure S1. M3258 does not inhibit mitochondrial serine proteases. **a**. Cleavage of Ac-WLA-amc (100 μM) by purified monomeric human ClpP (6 μM) was measured as described [81]. Data are averages±standard deviation of three technical replicates. **b**. LonP activity was measured using a modified method reported by Fishovitz et al [82]. The digestion of 100 μM of the fluorogenic peptide substrate containing 5% of FRETN 89-98 and 95% non-FRETN 89-98, by 400 nM of LonP (concentration reported as monomeric subunit) was conducted in the presence of 10 mM magnesium acetate, 1 mM ATP and 5 mM DTT in 50 mM HEPES buffer, pH 8.0 at 37°C. The increase in fluorescence signal was detected using excitation wavelength of 320 nm and emission wavelength of 420 nm. All assays were conducted at least in duplicates. **c**. FITC-labeled β-casein (0.1 μg/ml), prepared as described in [83], was incubated with 0.02 μg/ml purified recombinant human HtrA2 (R&D Systems, Cat #1458-HT) in 50 mM Tris-HCl, pH 8.0, for 30 min at 45°C, analyzed on a 10% NuPAGE gel using MES running buffer. FITC-casein was detected by fluorescent imaging.

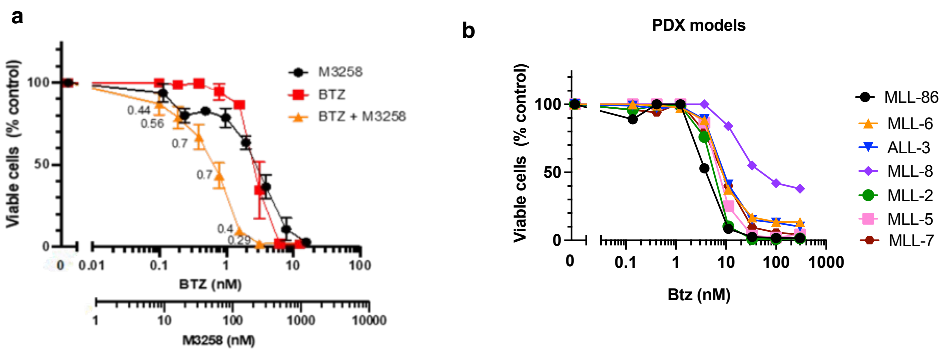

Figure S2. **a**. **Btz and M3258 cause synergistic cytotoxicity**. SEM cells were treated with Btz and M3258 (at a constant ratio) for 48 hrs, and viability was determined with Alamar Blue. Data are averages of two biological replicates. Error bars indicate standard error. Numbers on the graph are combination indexes determined by the CalcuSyn software. **b**. **MLL-AF4 expressing PDX models are Btz-sensitive *ex vivo***. Cells were treated *ex vivo* for 24 hrs and analyzed by Cell-Titer Glo. Numbers are average of 3-4 technical replicates.

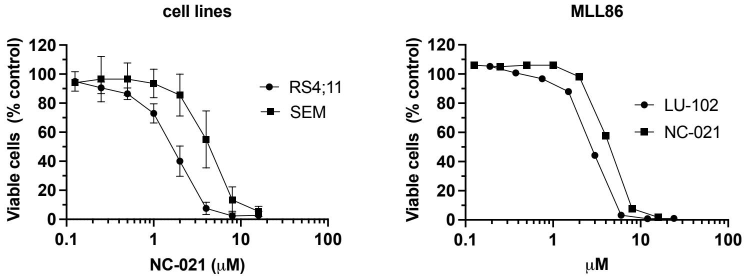

Figure S3. Single-agent activity of NC-021 and LU-102. **a**. SEM and RS4;11 cells were treated for 48 hrs and analyzed by Alamar Blue. Single agent activity of LU-102 is presented in Fig. 3d in [23]. **b**. MLL-86 cells were treated for 24 hrs are analyzed by CellTiter-Glo. Data on both graphs are averages of two biological replicates. Error bars indicate standard error.

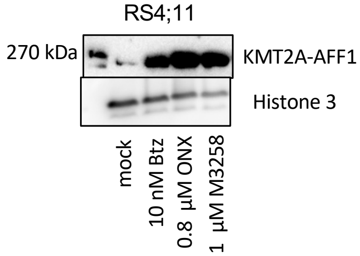

Figure S4. Treatment with Btz and IPIs upregulates KDMT2A-AFF1. RS4;11 cells were treated for 4 hrs, nuclear extracts were prepared, separated by SDS-PAGE, and transferred to a nitrocellulose membrane using methanol-free buffer and as described in [23]. Membranes were probed with rabbit polyclonal antibody against the N-terminal MLL1 (KMT2A) antigen (D2M7U, Cell Signaling Cat#14689; RRID:AB_2688009), and rabbit polyclonal antibodies against histone H3 (D2B12, Cell Signaling Cat#4620; RRID:AB_1904005).

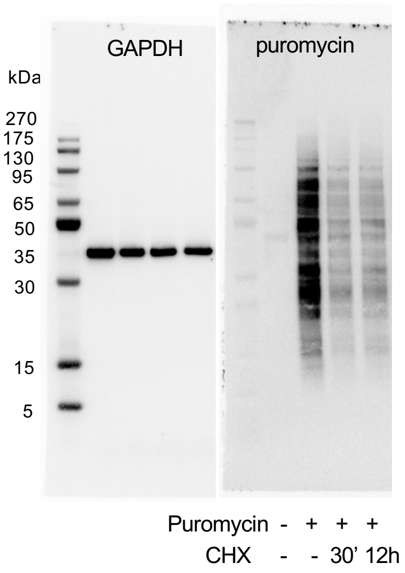

Figure S5. Inhibition of protein synthesis by CHX. Cells were treated with CHX (100 ng/ml) for times indicated, then spiked with puromycin for 15 minutes, harvested, lysed, and analyzed by western blot. Membrane was incubated simultaneously with mouse puromycin and rabbit GAPDH antibody, then with fluorescently labeled secondary antibodies, and imaged on two different channels. Uncropped images are shown.

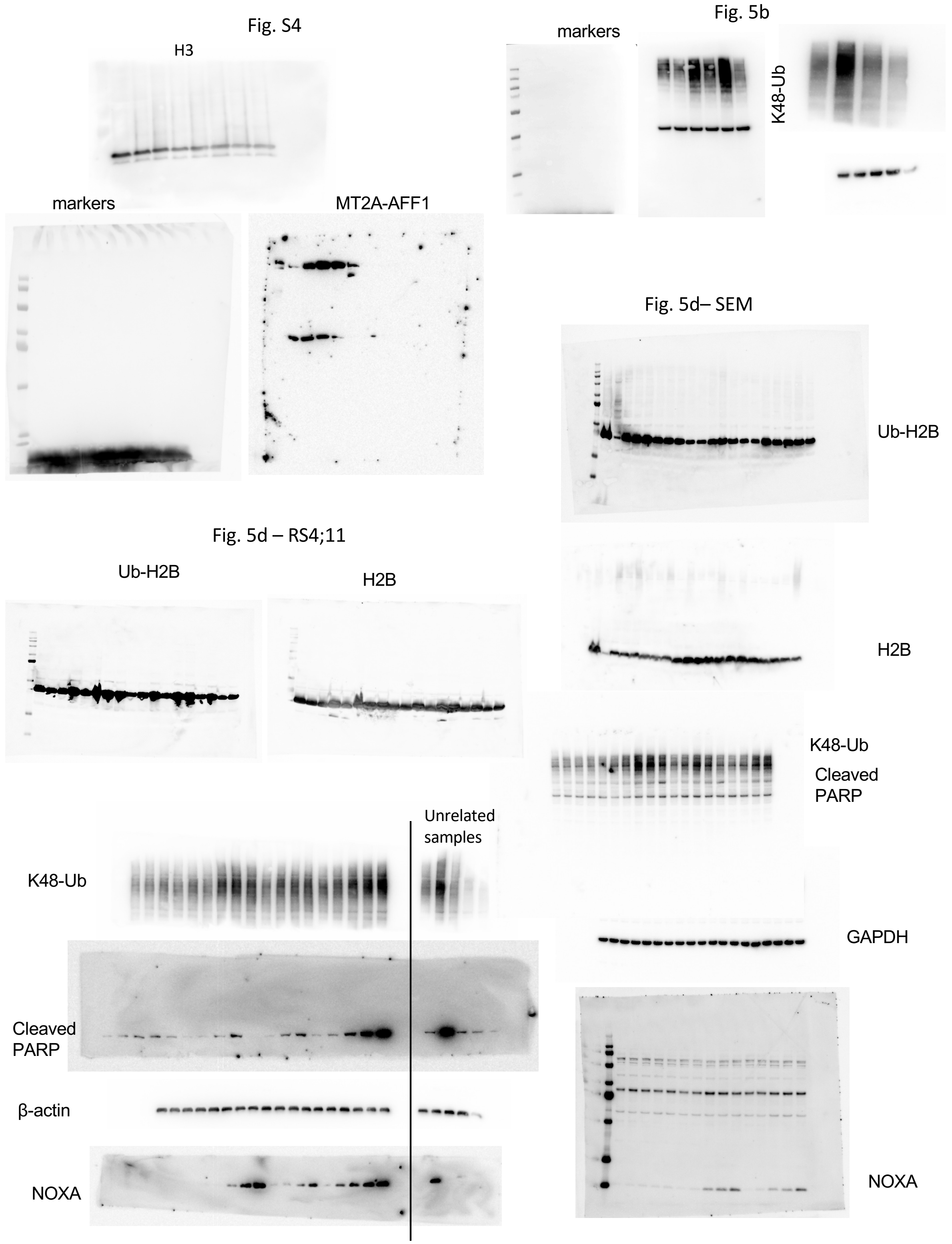

Figure S6. **Uncropped membranes**.

References

81. Wong KS, Mabanglo MF, Seraphim TV, Mollica A, Mao YQ, Rizzolo K, et al. Acyldepsipeptide Analogs Dysregulate Human Mitochondrial ClpP Protease Activity and Cause Apoptotic Cell Death. Cell Chem Biol 2018; 25: 1017-1030.e1019.
82. Fishovitz J, Li M, Frase H, Hudak J, Craig S, Ko K, et al. Active-site-directed chemical tools for profiling mitochondrial Lon protease. ACS Chem Biol 2011; 6: 781-788.
83. Akopian TN, Kisselev AF, Goldberg AL Processive degradation of proteins and other catalytic properties of the proteasome from *Thermoplasma acidophilum*. Journal of Biological Chemistry 1997; 272: 1791-1798.
